# Treatment of Teeth with Dead Pulps and Alveolar Abscess

**Published:** 1880-08

**Authors:** C. R. E. Koch

**Affiliations:** Chicago.


### ARTICLE IV.

### *Treatment of Teeth with Dead Pulps and Alveolar Abscess.*

BY DR. C. R. E. KOCH, OF CHICAGO.

Having at a very late day been asked to prepare a paper to be read before this society, and having very foolishly, though relucantly, consented to do so, to help the executive committee out of a scrape, I will endeavor in liquidation of that promise, to give you something which may serve as a starting point for discussion. My time and attention has been so much occupied of late with matters of various kinds, that it was impossible for me to prepare an essay.

If there are those among us who doubt that our profession has progressed in knowledge within the last twenty or twenty-five years, let them pause and compare the treatment of these cases referred to in the title of this paper, now, with that generally in vogue at that time. Then, an essay upon this theme might have been very briefly in two words, *extract them*, and would have met with the endorsement of the large body of the profession.

If now these cases are restored to usefulness, a great benefit is conferred upon humanity, and to do this with the least amount of suffering to the patient should be the aim of conservative practice.

It is true that failures in these cases sometimes occur to perplex and annoy us. Now and then a case may not progress just as we may wish or expect. In spite of precautions taken, our patient may call sometimes with an eye all but closed by a very much enlarged and rubicund cheek, and accompanied by a temper akin to that of a Sioux on the war path, exhibited in accents neither mild nor polite. Let us not be discouraged by such cases, but be all the more earnest in determining to know just what has caused this result and be careful tò avoid a *faux pas* of the same nature, if it may seem that the untoward condition was the result of mistake or neglect of ours. Sometimes such a result may occur, however, from causes far beyond our reach or recognition.

We may say it as a maxim, that all teeth with dead pulps in them, irrespective of the cause which destroyed their vitality, and regardless of whether they have produced annoyance, pain, periostitis or abscess, and whether decayed or not, should be treated; always provided that we have unmistakable evidence that the pulps are dead.

We might lay down the following general rules for the treatment of these cases:

First.—Cleanse the tooth and free the gum around it *from all irritating substances.*

Second.—Open the pulp-chamber, allowing any gases that may arise from decomposition to escape, and cleanse it and the root canals as thoroughly as possible.

The opening into the pulp chambers should always be made at a point where it will least weaken the tooth structure, that will give the most direct and free access to the introduction of the broach, medicaments and light, and that shall, when again filled up, least disfigure the tooth.

The practice of drilling holes into teeth that give indications of the presence of a dead pulp at right angles to their longitudinal axes and leaving these open, it is to be regretted is not yet obsolete. It is only rational treatment in part, and renders a permanent cure absolutely impossible.

True it permits the escape of gases arising from the decomposition and prevents their accumulation within the confined space, and consequent pressure upon and inflamation of the surrounding tissue, but it renders utterly impracticable the removal of the dead organic matter which, if done, would prevent the generation of these mephitic gases and hence preclude the necessity for an escapement flue, which latter must always be a nasty catch basin, in which are collected the tooth-destroying elements.

Third.—Do not plug up the canals tightly until you feel sure that there is no longer any danger from the formation of mephitic gases, and the pressure produced by their confinement within the walls of the canals.

The opening having been effected and the broken down tissue removed as thoroughly as possible, the walls should be thoroughly disinfected. Creosote, carbolic acid, or proof alcohol, will be all sufficient. If there has been pain, or soreness of the tooth, iodine should be freely exhibited in combination with the disinfectant, but unless an abscess has already been formed great care must be taken to introduce these remedies gently and without force. If introduced upon a broach with cotton, this should not be wound so tightly as to form a piston in the root canal.

Sometimes, indeed, it may be necessary in the treatment of these cases, in which there seems to be an indolent, sluggish periostitis which does not yield to the resolvent iodine treatment, to bring about an acute abscess, and there is no quicker way to produce it than to plug up the roots tightly.

The roots having been thoroughly disinfected, the tooth is given a trial, a little loose cotton being left in to prevent the ingress of other substances. It is rarely safe to seal up a tooth of this kind at the first sitting, and especially in cases where pulps have been dead for a long time, without giving patients any uneasiness; great care must be observed to avoid irritation of this nature. After a probationary period of a few days the cavity may be sealed up and the tooth again put upon probation.

Fourth.—Being satisfied that the root is ready to receive the filling, it should be filled with an indestructible material, which is sure to be carried to the apex of the root, and leave no open or air spaces along the walls.

The tooth having stood the test of being tightly sealed up for three or four days, or more, without any untoward signs, we may proceed to fill the root.

Most any substance employed for filling teeth is good enough in straight and regular shaped root canals, but in flat or compressed roots with tortuous canals, where it is at times impossible to know just what the broach has effected, there is nothing more adequate than gutta percha dissolved in chloroform. This should be pumped into the canals, after which a brief time should be allowed for the separation of a portion of the chloroform, which may be hastened by blowing a warm blast from the air syringe into the cavity. A piece of warmed gutta percha should then be crowded into the cavity until the root canal is filled. Another probationary period should then intervene before permanently filling the cavity over this. It is believed that this material will come nearer to being a perfect root filling than any other in these cases, because it can be carried with reasonable expectation of success to every part of the canals, and if any particles of the pulp should have escaped removal by reason of inaccessibility, it would be rendered impotent for mischief by reason of its becoming encased by the gutta percha solution. Of course in cases where there is an enlargement of the foramen it would not be a suitable filling.

Fifth.—Never fill a tooth permanently until a sufficient length of time has elapsed since filling the root, to leave a reasonable chance for expecting no evil results from the operation.

Sixth.—Have the patient to understand that the treatment of these cases of teeth with dead pulps in them are not simple, and that while there is no probability there is still a possibility of an abscess and suffering before the tooth shall be restored to usefulness.

In this consideration of the subject of dead pulps we do not consider pulps destroyed by ourselves as a necessary act, as in these cases there is generally no treatment necessary after the extraction of the pulp and before filling the root, although it is well even in such cases to defer the completion of the operation to another sitting.

Where the dead pulp has been allowed to go on in its mischief until periostitis has resulted in acute abscess, the pus should be carefully discharged through the opening in the tooth, and sufficient time should be allowed to have every particle drained off. When satisfied that no more is forming, which sometimes may take a week or more, creosote or carbolic acid and iodine should be forced into the canals so as to pass into and bathe the walls of the abscess, after which the case should be treated with the same caution already spoken of.

When abscess has become of long standing, and has established a fistulous opening upon the soft fissures through the alveolus, it is generally very easily cured. In such cases, after removal of debris from the root or roots, these should at once be injected with creosote and iodine, or carbolic acid, and if a free flowing of the medicine can be effected through the root and fistula, one application is generally sufficient, and the tooth may then be filled without much more treatment.

Occasionally we meet cases of this kind that, however, seem obstinate and do not yield to treatment readily, owing to the difficulty of forcing the remedies through the canals as in anterior roots of some lower, and buccal roots of upper molars. In such cases the medicine should be introduced through the fistula by means of an abscess syringe and the sulcus broken up. Generally the cases will yield to the remedies mentioned, but sometimes the aromatic sulphuric acid may be employed to good advantage.

Cases which do not yield to the treatment described occur sometimes, owing to the roughened and irritating condition of the apex of the root, which from long-continued bathing in the vicious secretions has become eroded; such cases call for surgical treatment, and may be met by trephining through the alveolar plate and excising the affected portion; or, by extracting the tooth, excising the end of root involved, polishing and replanting the tooth. These operations will not often be consented to by patients, and are only warranted in extreme cases.—*Trans. Illinois State Dental Society.*

